# TSH Receptor Antibody Functionality and Nomenclature

**DOI:** 10.3389/fendo.2017.00028

**Published:** 2017-02-15

**Authors:** George J. Kahaly, Tanja Diana

**Affiliations:** ^1^Molecular Thyroid Research Laboratory, Department of Medicine I, Johannes Gutenberg University Medical Center, Mainz, Germany

**Keywords:** Graves’ disease, functional TSH receptor antibodies, stimulating antibodies, blocking antibodies, nomenclature

This general commentary on the above, recently published *New England Journal of Medicine* review article wishes to clarify both the nomenclature as well as the role of autoantibodies (Ab) to the TSH receptor (TSH-R) pertaining to the serological diagnosis of Graves’ disease (GD).

Various terms have been used to describe the different types of TSH-R-Ab. It is important for the clinician to be aware of the different nomenclature as this will frequently reflect which assay is performed by the laboratory (Table 1). TSH-R-Ab, often referred to as TRAb, refers to any type of Ab interacting specifically with the TSH-R. Because these Ab are commonly assessed in a competitive binding assay, they are referred to as TSH-R-binding inhibitory immunoglobulins (TBII). By contrast, cell-based bioassays measure either TSH-R stimulatory antibodies (TSAb) or TSH-R-stimulating immunoglobulins, or alternately TSH-R-blocking antibodies (TBAb) or TSH-R-blocking immunoglobulins. Alternative terminologies for blocking antibodies are TSH-R-stimulating blocking Ab or TSH-R-blocking Ab (TRBAb). In this commentary, we will use TSH-R-Ab as a general term to refer to anti-TSH-R-Ab irrespective of the specific assay used. We will use TBII to refer to the Ab measured *via* binding assays, whereas Ab measured *via* bioassays will be referred to as TSAb for stimulatory and TBAb for blocking Ab.

**Table 1:** **Terminology for TSH receptor antibodies used in bioassays and binding assays**.

	Abbreviation
**Cell-based bioassay**	
TSH-R-stimulating antibodies TSH-R-stimulating immunoglobulins TSH-R-blocking antibodies TSH-R-stimulating blocking antibodies TSH-R-blocking immunoglobulins	TSAb TSI TBAb, TSB-Ab, or TRBAb TRBAb TBI
**Competitive-binding assay**	
TSH-R-binding inhibitory immunoglobulins	TBII

Graves’ disease is caused by persistent, unregulated stimulation of thyroid cells by TSH-R-stimulating Ab (TSAb) that activate the TSH-R (1). TSAb, like TSH, bind primarily to the large amino terminal ectodomain of the TSH-R and activate the cAMP signal transduction pathway leading to stimulation of thyroid hormone production and proliferation of thyrocytes. Since the discovery of TSAb as the causative agent of GD, there have been numerous studies that have demonstrated the significance of the levels of these Ab during the course of the disease as well as during antithyroid drug treatment in both adults and children (2, 3). Other types of TSH-R antibodies can antagonize or block the action of TSH and in doing so cause hypothyroidism in certain patients with various types of autoimmune thyroiditis, particularly Hashimoto’s thyroiditis. TSH-R antibodies that neither induce the cAMP signal pathway nor block the binding of TSH are referred to as neutral or recently “cleavage” Ab and currently are not known to have a functional effect (4). There is evidence, however, that neutral Ab may induce signaling pathways distinct from the cAMP pathway and may induce apoptosis (5).

As strongly recommended in the recently published hyperthyroidism guidelines of the American Thyroid Association (6), measurement of TSH-R-Ab is indicated both for the accurate and early diagnosis of autoimmune induced hyperthyroidism as well as during the management of patients with GD. Functional TSH-R-stimulating antibodies (TSAb) are causative of both the hyperthyroidism and the extra thyroidal manifestations of GD (7). TSAb can be sensitively and exclusively measured with validated bioassays that are available worldwide (8–11). In particular, the analytical performance and clinical utility of a FDA-cleared, stimulatory TSH-R bioassay in a large collective of patients with GD, both prior to as well as during medical antithyroid treatment, has been shown (12). In addition, a multicenter trial involving seven American and European academic referral centers confirmed the very high specificity, sensitivity, and positive and negative predictive values of this tool for the diagnosis of GD in children (13). Standardization and calibration of this bioassay, using a purely stimulatory human monoclonal TSH-R-Ab as international standard, allowed results to be reported in international units per liter (14). This has facilitated comparison of bioassay results with commercially available automated TSH-R-binding or TBII assays. A recent comparative study of seven immunoassays has shown that bioassays for TSH-R-Ab are more sensitive than the automated binding assays and exclusively differentiate between stimulatory and blocking Ab activity (15). Also, TSAb are a highly sensitive and predictive biomarker of the extra thyroidal manifestations of GD (16–18). Furthermore, the clinical relevance of the measurement of TSH-R-Ab and of TSAb in particular, during pregnancy in patients with autoimmune thyroid disease, was recently documented in a newborn with fetal/neonatal autoimmune thyrotoxicosis (19). Finally, incorporation and early utilization of TSAb into current diagnostic algorithms was shown to confer a 46% shortened time to diagnosis of GD and a cost savings of 47% (20).

## Author Contributions

The two authors listed have made substantial, direct, and intellectual contribution to the work and approved it for publication.

## Conflict of Interest Statement

TD has nothing to disclose. GK consults for Quidel, USA.
